# Genome Analysis and Physiology of *Pseudomonas* sp. Strain OVF7 Degrading Naphthalene and *n*-Dodecane

**DOI:** 10.3390/microorganisms11082058

**Published:** 2023-08-10

**Authors:** Anastasia A. Ivanova, Olesya I. Sazonova, Anton N. Zvonarev, Yanina A. Delegan, Rostislav A. Streletskii, Lidia A. Shishkina, Alexander G. Bogun, Anna A. Vetrova

**Affiliations:** 1 Federal Research Center “Pushchino Scientific Center for Biological Research of the Russian Academy of Sciences”, 142290 Pushchino, Russia; sazonova_oi@rambler.ru (O.I.S.); zvonarevibpm@gmail.com (A.N.Z.); mewgia@ya.ru (Y.A.D.); 2 State Research Center for Applied Microbiology and Biotechnology, 142279 Obolensk, Russia; kadnikova_lidiya@mail.ru (L.A.S.); bogun62@mail.ru (A.G.B.); 3 Laboratory of Ecological Soil Science, Faculty of Soil Science, Lomonosov Moscow State University, 119991 Moscow, Russia; streletskiyrostislav@mail.ru

**Keywords:** *Pseudomonas*, genome, naphthalene, *n*-alkane, biodegradation, biofilm

## Abstract

The complete genome of the naphthalene- and *n*-alkane-degrading strain *Pseudomonas* sp. strain OVF7 was collected and analyzed. Clusters of genes encoding enzymes for the degradation of naphthalene and *n*-alkanes are localized on the chromosome. Based on the Average Nucleotide Identity and digital DNA–DNA Hybridization compared with type strains of the group of fluorescent pseudomonads, the bacterium studied probably belongs to a new species. Using light, fluorescent, and scanning electron microscopy, the ability of the studied bacterium to form biofilms of different architectures when cultured in liquid mineral medium with different carbon sources, including naphthalene and *n*-dodecane, was demonstrated. When grown on a mixture of naphthalene and *n*-dodecane, the strain first consumed naphthalene and then *n*-dodecane. Cultivation of the strain on *n*-dodecane was characterized by a long adaptation phase, in contrast to cultivation on naphthalene and a mixture of naphthalene and *n*-dodecane.

## 1. Introduction

The rate of industrial development of cities contributes to the deterioration in the ecological condition of the environment. One of the main pollutants comprises various classes of hydrocarbons—products of crude oil conversion.

Polycyclic aromatic hydrocarbons (PAHs) are formed as a result of various biological processes [1] and are also products of incomplete burning of natural or anthropogenic sources (car exhaust gases, domestic heating, and cigarette smoke) [2]. These substances belong to the group of lipophilic organic pollutants, which are dangerous for the environment and human health [3]. Alkanes are saturated hydrocarbons that can have a linear (*n*-alkanes), cyclic (cycloalkanes), or branched (*iso*alkanes) structure. These hydrocarbon molecules are non-polar and chemically inert and have low solubility in water, making them difficult to metabolize by microorganisms. Such molecules have a tendency to accumulate in cell membranes and require additional energy to activate. However, some microorganisms (both aerobic and anaerobic) can use different types of hydrocarbons as the sole source of carbon and energy [4].

Some microorganisms have been found to be capable of degrading both PAHs and alkanes [5,6]. Moreover, the PAH and alkane degradation genes in microorganisms can be localized both on different plasmids [7] and on the same plasmid [8]. A total of 29 monooxygenase and 54 dioxygenase genes were found in the genome of *Paraburkholderia aromaticivorans* strain BN5. The biodegradation of different hydrocarbons, such as benzene, toluene, ethylbenzene, xylene, and naphthalene, can be linked to similar genes. It should be noted that of the sequences found in the *Paraburkholderia aromaticivorans* BN5 genome, six monooxygenase and fourteen dioxygenase genes are located on plasmids, and two alkane-1-monooxygenase (*alkB*) genes have chromosomal localization (CJU94_RS03555 on chromosome 1 and CJU94_RS23135 on chromosome 2) [9]. To date, there is little information on bacterial strains in which genes encoding PAH- and *n*-alkane-degrading enzymes have been detected simultaneously on the same chromosome [10,11].

Bacteria of the genus *Pseudomonas* are the most studied microorganisms capable of using hydrocarbons as a sole source of carbon and energy and producing bioactive substances that contribute to the absorption of such compounds. *Pseudomonas* are often used to study the biodegradation of various biogenic and xenobiotic pollutants. This is due to their metabolic universality and participation in various environmental processes.

Genomes of *Pseudomonas* strains belonging to different species have been characterized, e.g., *P. entomophila* (e.g., strain L48), *P. putida* (e.g., strains GB1, KT2440, F1, W619, S16, CSV86, SF1, DOT-T1E, B6-2, DLL-E4), *P. stutzeri* (e.g., strains KF716, A1501, CCUG29243), *P*. *protegens* (strain Pf-5), *P. fluorescens* (strains Pf-01, SBW25), *P. knackmussi* (strain B13), *P. aeruginosa* (e.g., strains PA7, PAO1, PACS2, 2192, and PA14), *P. pseudoalcaligenes* (strain KF707), *P. azelaica* (strains Aramco J, HBP1), *P. syringae* (strains B728a, DC3000 and 1448A,), and *P. mendocina* (strain YMP). The size of *Pseudomonas* genomes is about 6 Mb [12], and many genes are involved in the catabolism of different carbon sources and their adaptation to exist in a specific ecological niche.

In most cases, aromatic catabolism genes are organized into gene clusters that also contain specialized regulatory and transport genes and, in some cases, efflux pump genes [13]. For example, the naphthalene degradation genes, often used as a model substrate to study the various aspects of PAH catabolism, are organized into two operons in pseudomonads. Nine genes (*nahAaAbAcAdBFCED*) form the upper pathway operon (*nah* operon), which catalyzes the conversion of naphthalene to salicylate. The other 10 genes (*nahGTHINLOMKJ*) comprise the lower pathway operon (*sal* operon), which encodes the genes for the conversion of salicylate to tricarboxylic acid cycle intermediates by the enzymes of the catechol *meta*-cleavage pathway [8]. The *P. putida* AK5 strain is also known to contain the genes (*sgpGHIKGHB*) for the conversion of salicylate to tricarboxylic acid cycle intermediates via enzymes of the gentisate cleavage pathway [14]. Despite the fact that most naphthalene degradation genes identified in different bacteria have 99–100% identity with their analogs in other strains, the localization (plasmid or chromosome) and organization of these gene clusters may differ in each strain [15].

In the octane (OCT) plasmid of *Pseudomonas putida* strain GPo1, the alkane-degrading genes are organized as the *alkBFGHJKL* operon and are controlled by the products of another operon (*alkST*) located 40 bp downstream of the first operon [14]. van Beilen, J. B et al. [16] described a strain of *P. putida* P1 that contains the same operon structure (*alkBFGHJKL*), but in which *alkST* has been moved upstream of the operon and the *alkL* and *alkN* genes are not separated by the insertion sequence (IS) [15].

It is known that *Pseudomonas aeruginosa* strain PAO1 is capable of degrading *n*-alkanes, naphthalene, and phenanthrene [10,11,17,18]. Furthermore, the genes responsible for the degradation of the above substrates are located on the chromosome. The work of Qi, J. et al. [10] demonstrated the presence of functional genes of the naphthalene degradation pathway by gentisate in the PAO1 strain genome. Theo H.M. Smits et al. [11] demonstrated the presence of two alkane monooxygenase genes (*alkB1* and *alkB2*) homologous to the alkane monooxygenase gene (*alkB*) of strain P1. The same authors also showed the presence of functional rubredoxin reductase genes (*rubA1*, *rubA2*, and *rub*). Later [19], a cluster of *lao* genes was discovered, presumably involved in the degradation of primary long-chain alcohols. However, no detailed description of the *n*-alkane degradation pathway in *Pseudomonas aeruginosa* strain PAO1 has been found in the literature.

The process of microbial biodegradation of hydrocarbons involves adhesion to the substrate and the formation of biosurfactants/bioemulsifiers, biopolymers, solvents (e.g., acetone, ether, benzene), gases, and acids (e.g., stearic acid) to increase the bioavailability of hydrocarbon substrates. These substances also contribute to substrate detoxification [3,5,20].

The presence of oil hydrocarbons in the environment affects bacterial survival. Destruction of such substances often requires microbial adaptation to the pollutant, which is an important strategy for increasing the stress resistance and tolerance of microbial cells. Oil hydrocarbons, being toxic and persistent, act as a driver of natural selection in the microbial community, contributing to the evolution of degradation pathways. One example of microbial adaptation to extreme environmental conditions is the formation of biofilm [21]. A biofilm is an association of bacteria containing a slimy extracellular matrix with cells that can bind to hydrocarbons and enhance solubilization, leading to the degradation of these substrates. In addition, the hydrophobic nature of the bacterial surface has a direct effect on biofilm formation. Such an adaptation factor allows microorganisms to bind to surfaces of biotic and abiotic origin [22].

Despite the fact that many researchers have studied microorganisms that degrade oil hydrocarbons, very little work has been undertaken on the genomics and physiology of microorganisms capable of simultaneously degrading PAHs and *n*-alkanes. *Pseudomonas aeruginosa* PAO1, a multi-destructor of various hydrocarbons, has been the most extensively studied. However, this bacterium is pathogenic, making its use in environmental remediation technologies impossible. However, such multi-degrading strains can be used as model systems to study the mechanisms of simultaneous degradation of different classes of hydrocarbons. This is certainly important for the development and improvement of technologies for the bioremediation of oil-contaminated sites. 

The aim of the present study was to investigate the genetic and physiological characteristics of the *Pseudomonas* sp. strain OVF7 isolated from oil-contaminated soils of the “Pogranichnoye” oil field in Yamalo-Nenets Autonomous Okrug (Russia), which is capable of degrading both PAHs and *n*-alkanes and forming biofilms when grown in liquid media with carbon substrates.

A probably new bacterial species of the group of fluorescent pseudomonads, capable of forming biofilms having different architectures in the degradation of naphthalene, *n*-dodecane, and a mixture of these hydrocarbons, was isolated and is described herein. The simultaneous presence on the chromosome of gene clusters of the naphthalene degradation pathway via salicylate using the *meta*-pathway of catechol cleavage and gene clusters of the *n*-alkane degradation pathway via monoterminal oxidation was found. The genes encoding enzymes of the “classical” complete pathways of degradation of *n*-alkanes and naphthalene to Krebs cycle intermediates were described in the strain under study. A strain of *Pseudomonas* sp. strain OVF7 is interesting both as a model object for studying the mechanisms of degradation of different classes of hydrocarbons and as a promising agent for application in environmental clean-up biotechnologies.

## 2. Materials and Methods

### 2.1. Bacterial Strain

The strain OVF7 was isolated from the oil-contaminated soils of the “Pogranichnoye” oil field in the Yamalo-Nenets Autonomous Okrug (Russia). Strain OVF7 was stored in the collection of the Laboratory of Plasmid Biology (IBPM RAS (FRC PSCBR RAS), Pushchino, Russia). It is capable of utilizing *n*-alkanes, naphthalene, crude oil, and diesel fuel at 28 °C.

### 2.2. Chemicals

High purity (>98%) analytical-grade sodium succinate, dichloromethane, naphthalene, *n*-dodecane, *n*-octane, *n*-nonane, *n*-undecane, *n*-hexadecane, and *n*-decane were purchased from Sigma-Aldrich (St. Louis, MO, USA).

### 2.3. Genome Sequencing, Assembly and Annotation

A cetyltrimethylammonium bromide miniprep procedure [23] was used to isolate and purify total DNA from *Pseudomonas* sp. strain OVF7. Illumina and Oxford Nanopore technologies were used for DNA sequencing of *Pseudomonas* sp. strain OVF7. The FLAMING 106 flow cell (Oxford Nanopore Technologies (Oxford, UK) [ANT]) was used for sequencing on the MinION equipment. Using the ligation sequencing kit (catalog number SQK-L SK 109), libraries were prepared. In parallel, the S2 reagent kit (catalogue number 20012861; 2 × 100 bp) was used for nucleotide sequencing on the Illumina NovaSeq 6000 platform. The paired-end library was generated using the KAPPA Hyper Plus Kit (KAPA biosystems, Wilmington, MA, USA).

The programs SPAdes version 3.15.2 [24], Unicycler (May 2023) [25], and Flye 2.9 [26] were used to assemble the hybrid based on the Nanopore and Illumina reads. We used Pilon version 1.23 [27] and Bowtie2 version 2.3.5.1 [28] for error correction. To confirm that we had assembled a circular replicon, we analyzed the presence of overlapping ends. To make the results public, the data were deposited in the GenBank (BioProject, PRJNA987530; BioSample, SAMN35982525; GenBank, NZ_ CP128996).

Prokka software v1.14.5 helped us to annotate the genome [29]. We used the Basic Local Alignment Search Tool (BLAST) [30] to search for functions of some hypothetical proteins. 

Taxonomic analysis of the complete genome assembly was performed on the Type (Strain) Genome Server (TYGS) (https://tygs.dsmz.de (accepted date (2 June 2023)) [31], taking into account recently developed methodological updates [32]. The MASH algorithm [33] was used to search for genomes of closely related strains in comparison with the genome of *Pseudomonas* sp. OVF7. In addition, the 16S rDNA gene sequence was used to select 10 closely related type strains. Using RNAmmer [34] and BLAST [30], the 16S rDNA gene sequences of 19,149 type strains available in the TYGS database were extracted and compared with the same sequence in the genome of *Pseudomonas* sp. strain OVF7. According to the algorithm [35], the top 50 matching type strains were selected (according to the bit ratio). A final selection of the 10 closest type strain genomes was made using the distance formula d5 [35]. Digital DNA–DNA hybridization (DDH) values and confidence intervals were calculated using the recommended settings of GGDC 3.0 [32,36].

FASTME 2.1.6.1 [37] was used to construct a minimum evolutionary tree based on the intergenomic distances obtained. Tree rooting was performed at the midpoint [38] and visualized using https://itol.embl.de/ (accepted date (9 June 2023)) [39]. Species and subspecies were clustered according to [31] and [40], respectively. The OrthoANI algorithm [41] was used to calculate the average nucleotide genome identity (ANI) between strains of *Pseudomonas* sp. strain OVF7 and related strains.

The *rpoD* gene phylogenetic tree was built using the neighbor-joining method (BV-BRC) [42], accessed 25 May 2023. The CGView program [43] was used to image the chromosome map of *Pseudomonas* sp. strain OVF7.

### 2.4. Growth Media and Conditions

To prepare the inoculum, the cell culture was grown in liquid mineral medium containing 2% (*v*/*v*) succinate. The suspension obtained was centrifuged and the sediment was washed with physiological solution. The sediment was then resuspended in physiological solution and the cell concentration in the suspension was increased to 1 × 10^8^ CFU/mL using a turbidity standard. The inoculum was added to the test systems until the final concentration was about 1 × 10^6^ CFU/mL.

The *Pseudomonas* sp. strain OVF7 was grown in Erlenmeyer flasks containing 100 mL of mineral medium with the following composition [44]. The flasks were sealed with sterile rubber stoppers to prevent abiotic losses of the substrates tested. The plugs were opened in a sterile manner for 2–3 min daily to renew the gas phase, taking into account the amount of oxygen in the system. Succinate, naphthalene, phenanthrene, toluene, benzene, anthracene, *n*-octane, *n*-nonane, *n*-dodecane, *n*-undecane, *n*-dodecane, and *n*-hexadecane were used as carbon and energy sources to assess the substrate specificity of the strains. Substrates were added at 1 g/L. The strain was grown in flasks for 1–7 days at 28 °C and 180 rpm. Visual assessment of culture growth was performed throughout the period.

To plot the growth curve, a suspension was taken and diluted ten-fold, and then the suspension was seeded onto Lysogeny Broth (LB) agar medium [45]. Plates were incubated at 28 °C for 2 days. Individual colonies were then counted. Growth curves were constructed from the data obtained.

The loss of naphthalene, *n*-dodecane, and the mixture of naphthalene and *n*-dodecane was evaluated in the described systems at the end of the exponential growth phase. For the evaluation of abiotic losses of introduced hydrocarbons, systems without microorganisms were used for the same period of time. All results were derived from five independent replicates.

At the end of the exponential growth phase, *Pseudomonas* sp. strain OVF7 was also sampled for further microscopic studies. For microscopy, the culture grown in mineral medium with succinate as the sole source of carbon and energy at the end of the exponential growth phase was used as a control system.

### 2.5. Determination of Hydrocarbon Content in the Medium

Dichloromethane (1:1, *v*/*v*) was used to extract *n*-dodecane and naphthalene from the growth medium. Substrate content was estimated by gas chromatography (Agilent 6890, Agilent Technologies, Santa Clara, CA, USA) using a flame ionization detector. The chromatographic column was DB-1 (30 m × 0.25 mm id, 0.25 µm). The oven temperature was increased by 15 °C every minute (the initial temperature was 40 °C). The Collaborative Use Center, Department of Soil Science, and Lomonosov Moscow State University kindly provided equipment for sample analysis. Analytical standards were used for absolute calibration and quantification. The coefficient of correlation amounted to 0.99. ANOVA was *p* = 0.04. Samples were diluted 100-fold prior to analysis.

The degree of hydrocarbon biodegradation (D) was calculated using the following formula:

D = (C_0_ − C_i_)/C_0_ × 100 [%],

where C_0_ is the concentration of hydrocarbon in the experiment without microorganisms (abiotic control); C_i_ is the concentration of hydrocarbon in the experiment with microorganisms after i hours of growth (the end of the exponential growth phase).

### 2.6. Light and Fluorescent Microscopy

Cells were examined by phase-contrast and fluorescent microscopy in an AXIO Imager A1 (Zeiss, Oberkochen, Germany) with a filter set 56HE (Zeiss) at a wavelength of 490 nm (excitation) and 512 + 630 (emission). An Axiocam 506 (Zeiss) was used for image acquisition. Live and dead cells were revealed using a LIVE/DEAD™ BacLight™ Bacterial Viability Kit (Molecular Probes, Eugene, OR, USA). 

### 2.7. Scanning Electron Microscopy

The surface morphology of the biofilms was examined using scanning electron microscopy (SEM). Samples of the cells placed on membrane filters were fixed in glutaraldehyde vapor for 24 h at 4 °C and post fixed in OsO_4_ vapor for 3 h at 20 °C. After dehydration in propylene oxide vapor, the samples were coated with gold (Fine Coat Ion Sputter JFC-1100, Tokyo, Japan) and examined under a scanning microscope JSM-6510LV (JEOL, Tokyo, Japan).

## 3. Results

### 3.1. Nucleotide Sequence and Annotation

Sequencing and complete assembly of the genome of *Pseudomonas* sp. strain OVF7 revealed a circular chromosomal replicon of 7,174,656 bp (GC content: 60.35%). The chromosome contains 6628 coding sequences (CDS), 6 rRNAs, and 62 tRNAs. A function was assigned to 5147 CDS, of which 1480 CDS were annotated as hypothetical proteins. We identified genes involved in naphthalene and n-alkane degradation. We also found several genes for incomplete degradation pathways for caprolactam, toluene, biphenyl, xylene, benzoate, styrene, 2,4-dichlorobenzoate, atrazine, ethylbenzene, anthracene, biphenyl, and fluorobenzoate.

**Figure 1:**
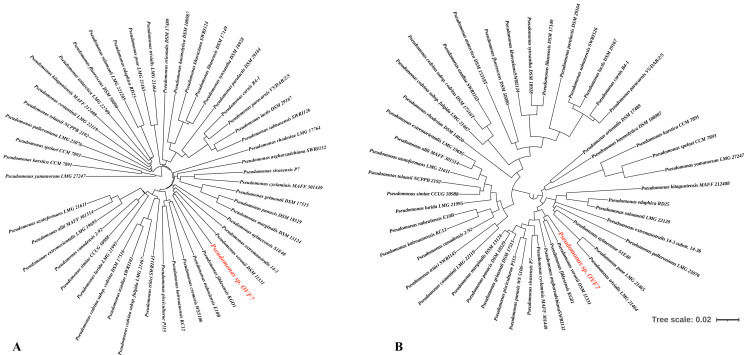
Phylogenetic trees of 49 type strains of *Pseudomonas* and the study strain OVF7. (**A**) Phylogenetic tree of the whole and draft genomes of the *Pseudomonas* type strains and *Pseudomonas* sp. OVF7. (**B**) Phylogenetic tree based on the *rpoD* gene. Maximum likelihood tree, GTR + G + I model (MEGA-X)) including, respectively, 49 type strains of *Pseudomonas* and the study strain, respectively. Bootstrap values were calculated on the basis of 1000 replications. The *Pseudomonas* sp. strain OVF7 is indicated in red font.

On the basis of the preliminary analysis of the 16S rRNA gene, the studied strain was assigned to the genus *Pseudomonas*, and specifically to the group of fluorescent pseudomonads. Based on the whole-genome sequencing, an attempt was made to determine the phylogenetic relationship between the studied strain and its closest type strains (Figure 1A). However, the whole-genome alignment did not allow an accurate determination of species affiliation. In accordance with the recommendations of Chun et al. [46], the Average Nucleotide Identity and digital DNA–DNA Hybridization values were used to compare the genome of *Pseudomonas* sp. strain OVF7 genome with those of closely related type strains. The comparisons of ANI and DDH for the strain *Pseudomonas* sp. strain OVF7 with these values for the strains of species of the group of fluorescent pseudomonads listed in Table S1 were 84.44–88.87% and 36.6–59.1%, respectively. Articles [36,46,47] suggest that when describing new species, these indices should be lower than 95% (ANI) and 70% (DDH) in comparison with type strains of known species.

According to the article by Girard L. et al. [48], the group of fluorescent pseudomonads includes the following species: *P. allii*, *P. antartica*, *P. asgharzadehiana*, *P. aylmerense*, *P. azadiae*, *P. azotoformans*, *P. canadensis*, *P. carnis*, *P. cedrina* subsp. *cedrina*, *P. cedrina* subsp. *fulgida*, *P. costantinii*, *P. cremoris*, *P. cyclaminis*, *P. edaphica*, *P. extremaustralis*, *P. carnis. cremoris*, *P. cyclaminis*, *P. edaphica*, *P. extremaustralis*, *P. extremorientalis*, *P. fildesensis*, *P. fluorescens*, *P. grimontii*, *P. haemolytica*, *P. kairouanesis*, *P. karstica*, *P. khavaziana*, *P. kitaguniensis*, *P. lactis*, *P. libanensis*, *P. lurida*, *P. marginalis*, *P. nabeulensis*, *P. orientalis*, *P. palleroniana*, *P. panacis*, *P. paracarnis*, *P. paralactis*, *P. paralactis*, *P. pisciculturae*, *P. poae*, *P. rhodesiae*, *P. salmasensis*, *P. salomonii*, *P. simiae*, *P. sivasensis*, *P. spelaei*, *P. synxantha*, *P. tolaasii*, *P. tritici*, *P. trivialis*, *P. veronii*, *P. yamanorum*. The authors recommended the estimation of the phylogenetic relationships of the strains on the basis of the *rpoD* gene (Figure 1B). However, even using this approach, it was not possible to determine the species assignment of the strain. It can be noted that, based on the trees presented (Figure 1A,B), the closest species to the studied strain are *P. veronii* and *P. fildesensis*. However, there are currently no whole-genome type strains of these two species. So, it is not possible to make a detailed comparison of genome structure using, for example, the Mauve method.

Thus, based on the combination of approaches used (phylogenetic tree of the whole/draft genomes of the *Pseudomonas* type strains, phylogenetic tree based on the *rpoD* gene, ANI and DDH indexes), the isolated microorganism can be classified as a new species of the group of fluorescent pseudomonads.

### 3.2. Genes Potentially Involved in n-Alkanes and Naphthalene Degradation

Analysis of the whole-genome sequence of strain OVF7 showed that its chromosome contains all the genes that, according to literature data [16], are necessary for the conversion of *n*-alkanes to acyl-CoA derivatives. These genes are located in a chromosome region of about 15 kb and are organized into the two “classical” operons, *alkST* and *alkBFGHJKL*. The mutual arrangement of the *n*-alkane oxidation gene clusters and the overall organization of this chromosome region in strain OVF7 are identical to those in the strains *P. veronii* strain 7–41 (CP089552.1) and *P. putida* strain P1 (GCA_001865225.1) [8,14]. Note that in the case of *P. veronii* strain 7–41, the *alk*-genes are plasmid localized, whereas in the *P. putida* strain P1, the genes are localized within the chromosome. In addition, the *n*-alkane degradation genes in the strain under study, as in the cases of *P. putida* strain P1 and *P. veronii* strain 7–41, are flanked by identical copies of the insertion sequence ISPpu4. In general, the region containing the *alk*-genes clusters and flanking their mobile elements occupies a region of about 23 kb.

The identity of the nucleotide and deduced amino acid sequences of the *n*-alkane oxidation genes with the corresponding sequences available in databases was analyzed using the BLAST online resource (accessed on 20 June 2023). These indices were 100% for almost all *alk*-genes of strain OVF7 with the corresponding sequences of the strains *P. veronii* strain VI4T1 (ASM202932v1), *P. veronii* strain 7–41 (pPCP7-41), and *P. putida* strain P1 (Table S2). The exception was the gene encoding long-chain-fatty-acid-CoA ligase, *alkK*, for which the amino acid sequence identity was 99%. The organization of the *n*-alkane degradation genes was studied in detail using the OCT plasmid of the *P. putida* strain GPo1 as an example. The identities of the deduced amino acid sequences of the *alk*-genes of the OVF7 strain with the corresponding sequences of the OCT plasmid ranged from 47% to 92%. Moreover, the highest value was observed for alkane 1-monooxygenase, AlkB, and the lowest for rubredoxin-1, AlkF (Table S2).

In the chromosome of strain OVF7, the naphthalene and salicylate degradation gene clusters (*nah* and *sal* genes) are located downstream of the *alkBFGHJKL* gene cluster. The distance between the *alk* and *sal* genes is about 7 kb. The genetic organization of the naphthalene biodegradation system in pseudomonads has been well studied in the *P. putida* strain G7 carrying the NAH7 plasmid [49,50,51]. All genes required for the conversion of naphthalene to Krebs cycle intermediates in the *P. putida* strain G7 have plasmid localization. It should be noted that with the development of sequencing technologies it has been found that in pseudomonads the *nah* genes have about 90% homology with the corresponding genes of the NAH7 plasmid [8,52,53]. To determine the degree of similarity between the nucleotide and deduced amino acid sequences of the naphthalene degradation genes of strain OVF7, we compared the *nah* gene clusters with the corresponding sequences of other bacteria, including pseudomonads, deposited in public databases. In strain OVF7, as in the case of plasmids pCP7-41 (CP089552.1), pND6-1 (AY208917.2), pDTG1 (AF491307.2), and NAH7 (NC_007926.1), the naphthalene degradation genes are organized into two operons: the *nah* operon and the *sal* operon. The *nah* operon contains nine genes (*nahAaAbAcAdBFCED*) encoding enzymes for the oxidation of naphthalene to salicylate. The *sal* operon includes genes (*nahGTHINLOMKJ*) necessary for the cleavage of salicylate to Krebs cycle intermediates. Unlike the above plasmids, the *nah* and *sal* operons of OVF7 are a chromosomally localization. The genes belonging to the *nah* operon occupy a chromosome region of 9 kb, whereas the size of the *sal* operon is 10 kb. It should also be noted that the *nah* and *sal* genes are transcribed in opposite directions from each other. The identities of the deduced amino acid sequences of the *nah* and *sal* operon genes of strain OVF7 with the corresponding sequences of the naphthalene catabolic plasmids NAH7 (NC_007926. 1), pND6-1 (AY208917.2), pDTG1 (AF491307.2), pCP7-41 (CP089552.1), and the unnamed plasmid of strain *P. veronii* strain Pvy (CP039632.3), were 72–100% (Table S2). The lowest similarity was shown with the catabolic genes of plasmid NAH7 and the highest similarity with pCP7-41. The identity of the deduced amino acid sequences of almost all *nah* and *sal* genes between OVF7 and pCP7-41 was 100%, except for the gene encoding the outer membrane beta-barrel protein (nahQ). It was 99% for NahQ. Clusters of *nah* and *sal* genes were also found in *P. veronii* strain VI4T1. The identity of the deduced amino acid sequences of the genes responsible for naphthalene catabolism in *P. veronii* strain VI4T1 and *Pseudomonas* sp. strain OVF7 was 100% for all genes. A CDS with a high degree of homology of the deduced amino acid sequence with the corresponding sequence of plasmid pCP7-41 (UHH01036.1) and the unnamed plasmid of *P. veronii* strain Pvy (CP039632.3) was found downstream of the *sal* operon (100% and 95%, respectively). This CDS encodes a LysR-type transcriptional regulator required for the expression of the *nah* and *sal* operons. The IS elements of the IS110 family transposase flank the *nah* operon of strain OVF7.

**Figure 2:**
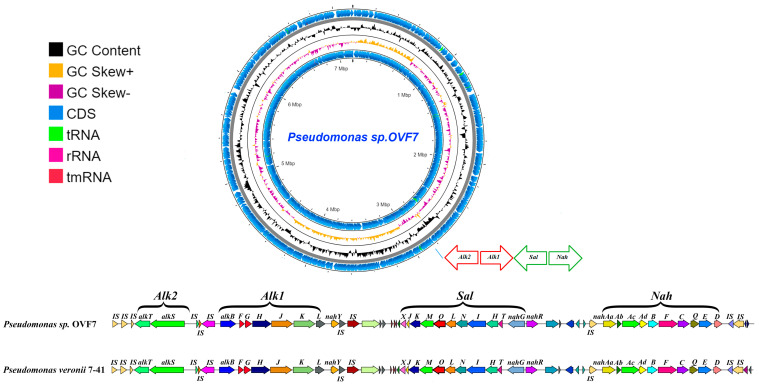
Circular map of the *Pseudomonas* sp. OVF7 chromosome and comparison of the *alk*–*sal*–*nah* region in strains *Pseudomonas* sp. OVF7 and *P. veronii* 7-41 (pCP7-41 plasmid). From outside to the center: all CDS and RNA genes on forward strand, GC skew, GC content, all CDS and RNA genes on reverse strand.

The mutual arrangement of the *n*-alkane and naphthalene degradation genes in the OVF7 strain chromosome is such that the *nah* genes are located downstream of the *sal* genes, while the *alk* genes are located upstream of the *sal* genes. The genetic organization of the *alk*–*sal*–*nah* region in the *Pseudomonas* sp. strain OVF7 chromosome is completely consistent with that of the *P. veronii* strain 7–41 (pCP7-41 plasmid) (Figure 2). In addition, the identity of the nucleotide sequences of these regions in both strains was 99%.

### 3.3. Growth of Pseudomonas *sp.* OVF7 in Mineral Medium with n-Dodecane and Naphthalene

The ability of *Pseudomonas* sp. strain OVF7 to grow at 28 °C in liquid mineral medium on different PAHs and *n*-alkanes was studied. As a result, the strain did not grow on the following substrates: phenanthrene, toluene, benzene, anthracene, and *n*-hexadecane. The strain OVF7 showed high activity in the medium containing naphthalene and during growth on *n*-alkanes (*n*-octane, *n*-nonane, *n*-decane, *n*-dodecane, and *n*-undecane). The formation of small flakes was observed when the strain grew in the culture medium containing *n*-octane, *n*-nonane, *n*-decane, and *n*-undecane. However, in the medium with *n*-dodecane as the sole source of carbon and energy, the formation of a dense layer of small bubbles was observed at the culture medium–hydrocarbon interface, while no flakes were observed in the remaining part of the medium. It was decided to use *n*-dodecane as a model *n*-alkane for further work. Strain OVF7 was also able to utilize a mixture of naphthalene and *n*-dodecane in a liquid mineral medium. To assess the abiotic loss of substrates (naphthalene and/or n-dodecane), an experiment was carried out in flasks containing hydrocarbons without microorganisms. Abiotic loss was practically not observed and was about 1–2% over 7 days because the flasks were sealed with rubber plugs.

**Figure 3:**
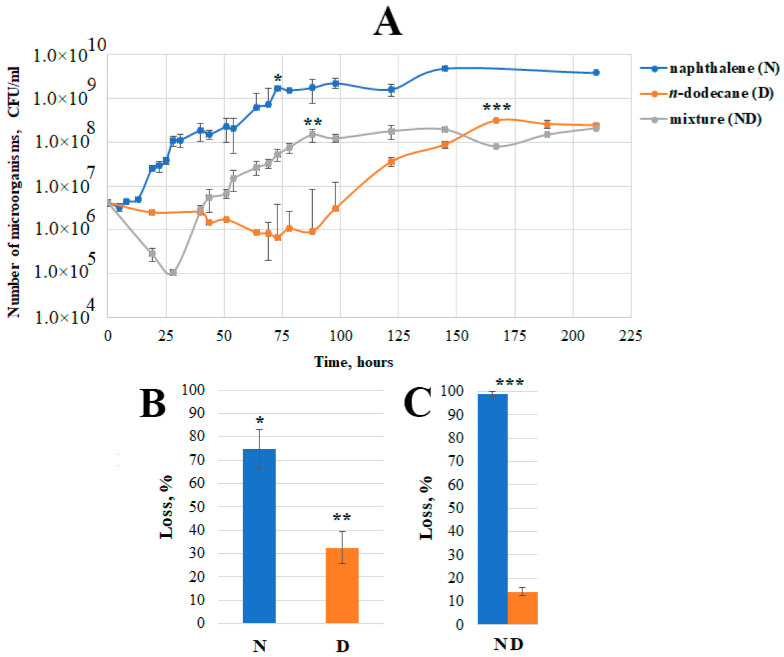
Physiological parameters of growing *Pseudomonas* sp. strain OVF7 in mineral medium with naphthalene (N), *n*-dodecane (D), and a mixture of naphthalene and *n*-dodecane (ND). (**A**)—Growth curves; (**B**)—Loss of naphthalene and *n*-dodecane in systems containing only one of these substrates; (**C**)—Loss of naphthalene and *n*-dodecane in a system containing a mixture of these substrates; *, **, *** on A denote the time of the end of the exponential growth phase corresponding to the naphthalene and/or *n*-dodecane loss data on (**B**,**C**).

Growth curves of *Pseudomonas* sp. strain OVF7 obtained by culturing the strain in a liquid mineral medium containing naphthalene (1 g/L), *n*-dodecane (1 g/L), and a mixture (naphthalene—*n*-dodecane—1 g/L (0.5 g/L each substrate)) were constructed (Figure 3).

As can be seen from Figure 3, when grown in the medium containing naphthalene at 28 °C, the lag phase of the culture was quite short (compared to the lag phase in the *n*-dodecane or a mixture of naphthalene and *n*-dodecane) and lasted until about 13 h. A small plateau was observed in the middle of the exponential growth phase (after 30 h of cultivation). This is probably related to the time of adaptation of the strain to the accumulated salicylate (intermediate of the naphthalene degradation pathway) and the beginning of its consumption. The accumulation of salicylate at this time was not investigated in the present work. However, it was previously shown [8] that in the middle of the exponential growth phase on naphthalene in pseudomonads there is a slight accumulation of salicylate and then its consumption. Such a phenomenon is necessary for the activation of *sal* operon genes. After 75 h of cultivation, the bacterium reached the stationary phase of growth and the number of microorganisms was about 10^9^ CFU/mL, which remained practically unchanged thereafter. At this time, the loss of naphthalene was 75%, i.e., the strain was metabolizing about 0.75 g of the substrate.

A long period of adaptation of the bacterial culture to the substrate (87 h) was observed when growing on *n*-dodecane (initial concentration 1 g/L) in liquid mineral medium at 28 °C. The exponential phase was monotonous and the culture reached its maximum abundance (10^8^ CFU/mL) at the end of the exponent after 167 h of cultivation. At this time, the loss of *n*-dodecane was 32%, i.e., the strain metabolized about 0.32 g of the substrate. It is likely that the lower consumption of *n*-dodecane by the strain influenced the lower maximum cell number compared to that when cultured on naphthalene.

A culture of *Pseudomonas* sp. strain OVF7 was also grown on a mixture of naphthalene and *n*-dodecane (with initial substance concentrations of 0.5 g/L naphthalene and *n*-dodecane, respectively) in liquid mineral medium at 28 °C. As shown in Figure 3, a lag phase was observed during the first 25 h of cultivation, which was accompanied by cell death and a decrease in number by more than an order of quantity. The subsequent exponential phase was similar in character to the growth on naphthalene. The secondary growth (after 50 h) began after a short period characteristic of salicylate accumulation (a similar phenomenon was observed in strain 7–41 when grown on a mixture of PAHs and *n*-alkane [8]). The stationary phase of growth started a little later than when growing on naphthalene as the sole of carbon and energy source (after 87 h). The maximum cell number was comparable to the maximum number when growing on *n*-dodecane alone (10^8^ CFU/mL). The degradation rate of naphthalene reached the value of 100% and that of *n*-dodecane was 12% (Figure 3), which was about 0.56 g/L of carbon substrates.

It can be concluded that the mixture of hydrocarbons (naphthalene–*n*-dodecane) had a toxic effect on the bacterial culture during the first hours of cultivation of *Pseudomonas* sp. OVF7. After the adaptation period, the character of the growth curve was as close as possible to the growth curve obtained when the strain was cultured on naphthalene alone.

### 3.4. Microscopic Data

In addition, the cell precipitate was surrounded by a slime-like substance characteristic of the biofilm matrix. To confirm the ability to form biofilms during growth on different substrates, the *Pseudomonas* sp. strain OVF7 was cultured in liquid mineral medium containing succinate (as a control), naphthalene, *n*-dodecane, and a mixture of naphthalene and *n*-dodecane. The cultured cells were examined by phase-contrast, fluorescence, and scanning electron microscopy.

**Figure 4:**
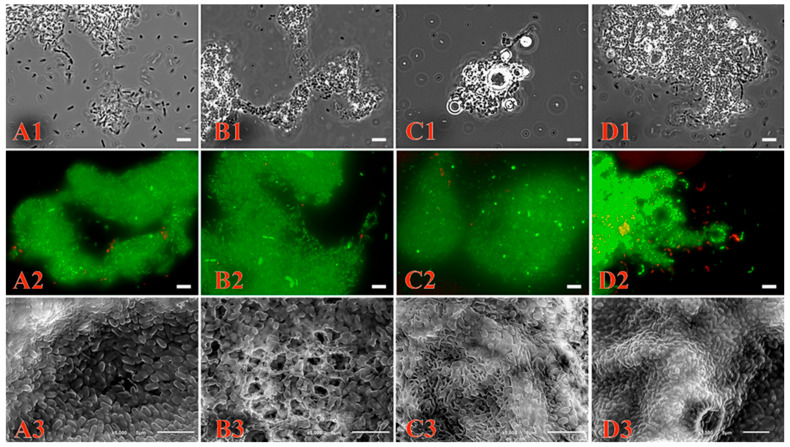
Biofilm formation of *Pseudomonas* sp. OVF7 when grown in liquid mineral medium with succinate (**A1**–**A3**), naphthalene (**B1**–**B3**), naphthalene–*n*-dodecane mixture (**C1**–**C3**) and *n*-dodecane (**D1**–**D3**), scale bar—5 µm. (**A1**,**B1**,**C1**,**D1**)—images obtained by phase-contrast microscopy; (**A2**,**B2**,**C2**,**D2**)—images obtained by fluorescence microscopy; (**A3**,**B3**,**C3**,**D3**)—images obtained by scanning electron microscopy.

As shown in Figure 4, *Pseudomonas* sp. strain OVF7 formed a biofilm. Moreover, the architecture of the biofilm depended on the substrate (Figure 4(A3,B3,C3,D3)). When cultured in liquid mineral medium with succinate, the bacterial cells lined up side by side, forming a dense homogeneous layer (Figure 4(A3)). The strain formed a biofilm whose matrix was perforated by a system of microchannels when naphthalene was used as the sole source of carbon and energy (Figure 4(B3)). The biofilm had a porous spongy structure when the *Pseudomonas* sp. strain OVF7 was in a bi-substrate system (naphthalene and *n*-dodecane) (Figure 4(C3)). The use of *n*-dodecane as the sole substrate resulted in the formation of a dense biofilm layer (Figure 4(D3)).

In addition, images obtained by fluorescence microscopy (Figure 4(A2,B2,C2,D2)) showed the formation of biofilms from living cells.

## 4. Discussion

*Pseudomonas* has been actively studied since 1894. These strains are among the most common genera in different microbial communities. Therefore, these microorganisms are responsible for various ecological functions. The ability of *Pseudomonas* to degrade various hazardous organic pollutants is well known [54]. Such metabolism is due to both the plasticity of the microbial genome and the presence of horizontal gene transfer as one of the means of bacterial adaptation to anthropogenic environmental pollutants. A better understanding of the genomic architecture and dynamics of bacterial genomes is necessary for the successful development of strategies for bioremediation of contaminated sites and water areas.

*Pseudomonas* have a large number of assemblies represented in various databases such as NCBI, BV-BRS, TYG, and *Pseudomonas*.com [31,42]. A detailed taxonomy of this genus is still lacking and, despite its long history of study and extensive databases, is constantly being updated with new results. Analysis of the 16S rRNA gene currently allows only the genus identity of *Pseudomonas* to be determined with certainty. The modern development of whole-genome sequencing technologies has greatly expanded the base of publicly available genomes. The analysis of genomes, or more precisely, their comparison using the ANI and DDH indices [36,54,55,56,57], has allowed a better identification of bacterial taxa. In our case, a whole-genome sequence (Figure 1A) analysis of the above-mentioned indices of the *Pseudomonas* sp. strain OVF7 (Table S1) in comparison with type representatives of the genus *Pseudomonas* did not allow its species identity to be determined. Based on the TYG server data, the strain was only identified as belonging to the group of fluorescent pseudomonads. Girard L. et al. [48] performed a large-scale study of the phylogeny of different *Pseudomonas* species based on the *rpoD* gene and whole genome sequences. The authors proposed to use the comparison of *rpoD* gene sequences as one of the representative approaches for species classification of *Pseudomonas*, including the group of fluorescent pseudomonads. Based on the *rpoD* gene (Figure 1B), it was also not possible to determine the species identity of *Pseudomonas* sp. strain OVF7. Only the closely related species *P*. *veronii* and *P*. *fildesensis* were detected. Moreover, the synergism of the presented approaches allows us to assume that the strain studied in the present work is a member of a new species of *Pseudomonas*. Therefore, our future plans include a detailed polyphasic taxonomy using phenotypic, chemotaxonomic, genetic, and phylogenetic methods to confirm or refute this hypothesis.

Analysis of the chromosome of *Pseudomonas* sp. strain OVF7 revealed the presence of clusters of genes encoding enzymes for the biodegradation of *n*-alkanes and PAHs (Figure 2). The nucleotide and amino acid sequence similarity of these genes to similar sequences described for destructor strains of the genera *Pseudomonas* should be noted. In addition, the presence of IS elements at the ends of degradative operons indicates a possible origin of catabolic genes in the genome of the strain under study by horizontal gene transfer from a strain with a similar organization of xenobiotic biodegradation pathways. The presence of IS elements may indicate that the strain under study has potential for application in various technologies of environmental remediation of crude oil and oil products.

Based on analyses of the OVF7 strain genome and data from literature studies, we inferred the pathways of naphthalene and *n*-dodecane metabolism, which are shown in Figures S1 and S2, respectively. Potential genes encoding degradation enzymes for these compounds are presented in Table S2. The multi-degrader *Pseudomonas aeruginosa* strain PAO1 is currently being studied in detail. The genome of *Pseudomonas* sp. strain OVF7 contains genes for naphthalene catabolism via the meta-pathway of catechol cleavage to Krebs cycle intermediates. Strain PAO1 also has a complete pathway for naphthalene degradation, but via gentisate. The ability of strain PAO1 to degrade *n*-alkanes is also known; several homologues of genes responsible for the initial steps of the degradation pathway (monooxygenases and rubredoxins) have been found, but further steps of degradation of the formed metabolites are still under investigation. For example, it is not yet clear whether there is a complete degradation pathway from *n*-alkanes to Krebs cycle intermediates in strain PAO1. In the studied strain OVF7, the structure of the genes responsible for the degradation of aliphatic compounds is organized into two “classical” operons of the complete pathway of degradation of *n*-alkanes to Krebs cycle intermediates (a similar structure was found in the OCT plasmid). Since strain PAO1 belongs to pathogenic species, it can only be considered as a model for the study of degradation mechanisms and its practical application is not possible.

As the closest species to the OVF7 strain under investigation were *Pseudomonas veronii* and *Pseudomonas fildensis*, representatives of the group of fluorescent pseudomonads, we decided to analyze the degradative characteristics of strains of these species in the literature. Pavlov M.S., et al. first described a new species of *P*. *fildesensis* [58]. However, no literature sources describing hydrocarbon-degrading strains of the species *Pseudomonas fildesensis* have been found to date. In contrast, the species *Pseudomonas veronii* is known for its metabolic potential against hydrocarbons such as alkyl methyl ketones (*Pseudomonas veronii* strain MEK700 [59]); pentachlorophenol (*Pseudomonas veronii* strain PH-05 [60]); benzene and toluene (*Pseudomonas veronii* strain 1YdBTEX2 [61,62]); phenol (*Pseudomonas veronii* strain Ju-A1 [63]); 2,4,6-trinitrotoluene (*Pseudomonas veronii* strain S94 [64]); dibenzo-p-dioxin, dibenzofuran, and chlorodibenzo-p-dioxins (*Pseudomonas veronii* strain PH-03 [65]); 4-amylphenol and 4-hexylphenol (*Pseudomonas* strains INA04, INA05, and INA06 [66]); chlorobenzene (*Pseudomonas veronii* strain UFZ B549 [67]); 4-chorosalicylate (*Pseudomonas veronii* strain MT4 [68]); *Pseudomonas veronii* strain B547 [69], *Pseudomonas veronii* strain UFZ B549 [70,71], and *Pseudomonas veronii* strain 16-6A [72]); and naphthalene and *n*-decane (*Pseudomonas veronii* strain 7–41 [8]). It should be noted that the level of naphthalene degradation in strain 7–41 was comparable to that of the studied strain OVF7 (about 70%).

The number of studies on the degradative properties of microorganisms is increasing every year. Such studies are important for the development of bioremediation approaches. However, the degradation mechanism of oil hydrocarbons by both individual strains and by their consortia is still the subject of active studies. The uniqueness of the strain studied lies in its multi-degradation ability with respect to different classes of hydrocarbons. Therefore, at the stage of the physiological characterization of the strain, we tried to extrapolate a model experiment with a mixture of the different classes of hydrocarbons to the conditions under which microorganisms degrade such complex compounds as crude oil and oil products. There is no doubt that in such complex substances there are various physico-chemical interactions and the mutual influences between different classes of hydrocarbons on each other. Such chemical processes may also have a direct influence on the mechanism of pollutant degradation. The toxic effects of most hydrocarbons are due to a general, non-specific effect on membrane fluidity due to their accumulation in the lipid bilayer, causing an increase in membrane fluidity, resulting in non-specific membrane permeability. Most compounds with high hydrophobicity, such as alkanes, PAHs, and biphenyls, have very low solubility in water, so their bioavailability is too low to exhibit toxic effects. [73]. However, we observed a specific effect of bacterial cells sticking together as aggregates when cultured in an aqueous medium containing a hydrophobic carbon source. To rule out possible toxic effects of xenobiotics on cells and to determine cell viability, a live/dead cell detection method was applied using the LIVE/DEAD™ BacLight™ Bacterial Viability Kit. This fluorescence microscopy method assesses the viability of bacterial populations based on the integrity of the cell membrane. Cells with damaged membranes, considered dead or dying, stained red, whereas cells with intact membranes stained green. Since almost the entire cell population appeared to be alive under fluorescence microscopy regardless of the carbon source, and scanning microscopy clearly showed an organized structure, we suggest that the formation of trophic bonds is a feature of this strain and not the result of toxic effects of xenobiotics. Such bacterial structures and the reconstruction of their organization on a hydrophobic carbon source have been studied in detail by Dmitriev, V.V. [74].

In the present work, it was shown that *Pseudomonas* sp. strain OVF7 is able to grow on aliphatic substrates of the C8-C12 series and on naphthalene, and data on the degradation of naphthalene, *n*-dodecane, and their mixture (Figure 3) were obtained. We assume that the catabolic operons observed in the course of this work (described in Section 3.2) are responsible for the degradation of naphthalene and *n*-dodecane in the genome of *Pseudomonas* sp. strain OVF7. Our assumption is based on the high similarity of the structures of these operons (order and arrangement of genes, similarity of nucleotide sequences of regions containing *nah*, *sal*, and *alk* genes, and similarity of deduced amino acid sequences) with the operons for the degradation of *n*-alkanes and naphthalene, for which the functionality of degradation genes has been confirmed [8,14,51,52]. In addition, the differences in the lag phase shown on the bacterial growth curves (Figure 3) suggest different toxicity and bioavailability of different classes of hydrocarbons. It is important to note that the mixture of hydrocarbons contributes to the change in the growth curve, especially in the early stages. We think that this is due to the intermolecular interactions between *n*-dodecane and naphthalene [75,76]. Such a phenomenon is likely to influence the toxicity and bioavailability of the hydrocarbon mixture with respect to the strain studied. Summarizing the results of the physiology of the studied strain during the experiments, it can be assumed that *Pseudomonas* sp. strain OVF7 is capable of more active naphthalene degradation compared to *n*-dodecane, which correlates with the research data of the *Pseudomonas veronii* strain 7–41 of Mullaeva et al. [8]. However, in contrast to the above bacterium, no strain death was observed in the bi-substrate system. Perhaps such an effect is due to the ability of the strain to synthesize biopolymers and form a biofilm.

Biofilm formation is a sequential process controlled by cellular, surface, and environmental factors. To date, the mechanisms and components of biofilms formed by *Staphylococcus epidermidis* have been well studied. It has been found that most *S. epidermidis* strains are capable of forming so-called *ica*-dependent biofilms. The main component of *ica*-dependent biofilms is a polysaccharide-polymer β-1,6-N acetylglucosoamine called polysaccharide intercellular adhesin (PIA). The *icaABCD* operon is responsible for the synthesis of PIA. It consists of four genes: *icaA* (1238 bp), *icaD* (305 bp), *icaC* (869 bp), and *icaB* (1238 bp), and is regulated by the repressor gene *icaR*. The *icaA* gene encodes the membrane protein IcaA, which has N-acetylglucosamine transferase activity. IcaA, with the participation of the IcaD protein, synthesizes PIA from uridine diphosphate N-acetylglucosamine [77]. Another known PIA is PNAG (poly-β-1,6-N-acetyl-D-glucosamine). The *pgaABCD* operon is responsible for its synthesis in *Escherichia coli*, for example. Functionally and genetically related loci are found in other Gram-negative bacteria, including *Klebsiella pneumoniae*, *Yersinia* spp., *Bordetella* spp., *Pseudomonas fluorescens*, *Actinobacillus pleuropneumoniae*, *Burkholderia cepacian*, and *Aggregatibacter actinomycetemcomitans*. PNAG production by *E. coli*, *A. pleuropneumoniae*, *Acinetobacter baumannii*, *Bordetella* spp., *A*. *actinomycetemcomitans*, and *Yersinia pestis* has been confirmed biochemically and/or immunologically [78]. 

The strain *Pseudomonas* sp. strain OVF7 was able to form biofilms when grown in mineral medium with the following substrates as carbon and energy sources: naphthalene, succinate, *n*-dodecane, and a mixture of naphthalene and *n*-dodecane. The genome of the *Pseudomonas* sp. strain OVF7 strain, which we studied using the Bacterial and Viral Bioinformatics Resource Center (BV-BRC) service, revealed the absence of the *icaABCD* operon (or its homologues) and the presence of the *pgaABCD* locus. The locus *pgaABCD* has a high degree of similarity to the *Burkholderia cepacia* strain J2315 and *B. cepacia* strain ATCC25416 genetic locus [78], which encodes proteins involved in the biosynthesis and export of PNAG.

In [79], it was shown that *P. fluorescens* biofilm growth was affected by cultivation conditions (semi-static or dynamic) and changes in nutrient availability. The authors suggested that the observed differences in biofilm formation represent a specific response of the bacteria to nutrient availability and composition. Dynamic conditions with high nutrient levels resulted in reduced biofilm formation, described by heterogeneously distributed clusters of cells. Biofilms grown at lower nutrient levels and high flow rates were fully developed flat homogeneous biofilms. Biofilms growing in nutrient-rich media produced more exopolymers, resulting in a significant elastic response, as determined by high biofilm viscosity. More rigid biofilm properties, characterized by a higher modulus of elasticity, were observed in biofilms grown in low nutrient conditions. The observed biofilm stiffness may be related to the lower levels of exopolymeric substances formed. When grown on naphthalene, the *Pseudomonas* sp. strain OVF7 formed a perforated biofilm, probably due to the need for more oxygen in the PAH biodegradation process compared to alkanes.

The present work demonstrates the adaptation features of *Pseudomonas* sp. strain OVF7 with respect to hydrocarbons, i.e., the formation of biofilms of different architectures, the large number of IS elements, and the presence in the genome of degradative operons responsible for the degradation of different classes of hydrocarbons.

## 5. Conclusions

The *Pseudomonas* sp. strain OVF 7 was classified as a group of fluorescent pseudomonads on the basis of whole-genome sequencing, ANI and DDH, and *rpoD* nucleotide sequence analysis, and is probably a member of a new species. Such a hypothesis will be further verified by a detailed polyphasic taxonomy using phenotypic, chemotaxonomic, genetic, and phylogenetic methods. The *Pseudomonas* sp. OVF 7 has multi-degradation properties against hydrocarbons of different classes (aliphatic and aromatic compounds) and is able to form biofilms. The biofilm architecture is different when the bacterium is grown in a liquid mineral medium with various carbon substrates. The latter probably shows the adaptive functions of the studied strain’s biofilm, including the resistance to toxic hydrocarbons in the process of their biodegradation.

## Data Availability

The data was submitted to the GenBank database under the following accession numbers: BioProject, PRJNA987530; BioSample, SAMN35982525; GenBank, NZ_ CP128996.

## Supplementary Materials

The following supporting information can be downloaded at: https://www.mdpi.com/article/10.3390/microorganisms11082058/s1, Table S1: List of type strains used in this study. ANI and DDH values of the *Pseudomonas* sp. OVF7 and related type strains, Table S2: Similarity of predicted gene products from the *nah*, *sal*, and *alk* gene clusters of *Pseudomonas* sp. OVF7 to selected homologs, Figure S1: Scheme of naphthalene catabolic pathway via catechol (red arrows) and gentisic acid (black arrows), Figure S2: Scheme of n-alkane catabolic pathway.
