# Supplementary material for: Genome Analysis and Physiology of Pseudomonas sp. Strain OVF7 Degrading Naphthalene and n-Dodecane
Source: Microorganisms. 2023 Aug 10;11(8):2058. doi: 10.3390/microorganisms11082058 (PMC10458186; doi:10.3390/microorganisms11082058)
Supplement: Supplementary file 1 [file microorganisms-11-02058-s001.zip › microorganisms-2506295-supplementary.pdf]

microorganisms

Supplementary Materials

Genome analysis and physiology of *Pseudomonas* sp. strain OVF7 degrading naphthalene and *n*-dodecane

Anastasia A. Ivanova[1,*], Olesya I. Sazonova[1], Anton N. Zvonarev[1], Yanina A. Delegan[1,2], Rostislav A. Streletskii[3], Lidia A. Shishkina[2], Alexander G. Bogun[2], and Anna A. Vetrova[1,*]

[1] Federal Research Center "Pushchino Scientific Center for Biological Research of the Russian Academy of Sciences", 142290 Pushchino, Moscow Region, Russia; mrs.ivanova.a.a@gmail.com (A.A.I.); sazonova_oi@rambler.ru (O.I.S.); zvonarevibpm@gmail.com (A.N.Z.); phdvetrova@gmail.com (A.A.V.);
[2] State Research Center for Applied Microbiology and Biotechnology; 142279, Obolensk, Russia; mewgia@ya.ru (Y.A.D); kadnikova_lidiya@mail.ru (L.A.S.); bogun62@mail.ru (A.G.B.);
[3] Faculty of Soil Science, Laboratory of Ecological Soil Science, Lomonosov Moscow State University; 119991 Moscow, Russia; streletskiyrostislav@mail.ru (R.A.S.).
* Correspondence: phdvetrova@gmail.com and mrs.ivanova.a.a@gmail.com (A.A.I.)

Table S1. List of type strains used in this study. ANI and DDH values of the Pseudomonas sp. OVF7 and related type strains.

№	Species	Accession number	Strain	DDH, %	ANI, %
1.	*P. allii*	GCA_013392005	MAFF 301514	55.6	87.74
2.	*P. antartica*	GCF_900103795	LMG 22709	48.1	87.27
3.	*P. asgharzadehiana*	GCA_019139815	SWRI132	53.6	87.34
4.	*P. aylmerense*	GCA_001702265	S1E40	51.6	88.20
5.	*P. azadiae*	GCA_019145355	SWRI103	48.3	87.36
6.	*P. azotoformans*	GCA_900103345	LMG 21611	54.9	87.86
7.	*P. canadensis*	GCF_000503215	2-92	42.4	87.57
8.	*P. carnis*	GCA_902329575	B4-1	47.3	86.91
9.	*P. cedrina* subsp. *cedrina*	GCF_001983175	DSM 17516	48.9	87.34
10.	*P. cedrina* subsp. *fulgida*	GCA_016937595	LMG 21467	41.3	87.45
11.	*P. costantinii*	GCF_001870435	LMG 22119	49.8	87.41
12.	*P. cremoris*	GCF_014230465	WS5106	50.8	87.10
13.	*P. cyclaminis*	GCA_015163715	MAFF 301449	51.4	87.85
14.	*P. edaphica*	GCA_005863185	RD25	44.3	87.10
15.	*P. extremaustralis*	GCA_000242115	14-3	50.0	88.17
16.	*P. extremorientalis*	GCF_001870465	LMG 19695	55.4	87.74

17.	*P. fildesensis*	GCA_001050345	KG01	57.2	88.63
18.	*P. fluorescens*	GCA_001269845	DSM 50090	51.3	87.33
19.	*P. grimontii*	GCF_900101085	DSM 17515	58.7	88.79
20.	*P. haemolytica*	GCF_009659625	DSM 108987	47.0	87.49
21.	*P. kairouanesis*	GCA_004682055	KC12	49.1	87.06
22.	*P. karstica*	GCA_009707515	CCM 7891	36.6	84.44
23.	*P. khavaziana*	GCA_019145205	SWRI124	46.5	86.86
24.	*P. kitaguniensis*	GCF_009296165	MAFF 212408	41.2	86.57
25.	*P. lactis*	GCF_001439845	DSM 29167	45.0	87.12
26.	*P. libanensis*	GCF_001439685	DSM 17149	48.6	87.05
27.	*P. lurida*	GCA_002563895	LMG 21995	55.1	87.60
28.	*P. marginalis*	GCA_007858155	DSM 13124	58.2	88.87
29.	*P. nabeulensis*	GCA_004682045	E10B	46.7	87.09
30.	*P. orientalis*	GCF_001439815	DSM 17489	47.4	87.34
31.	*P. palleroniana*	GCA_003031675	LMG 23076	50.6	86.70
32.	*P. panacis*	GCA_007858175	DSM 18529	59.1	87.09
33.	*P. paracarnis*	GCA_904063055	V5/DAB/2/5	46.9	86.72
34.	*P. paralactis*	GCF_001439735	DSM 29164	45.1	86.77
35.	*P. pisciculturae*	GCF_015461805	P115	50.4	86.71
36.	*P. poae*	GCA_001730605	LMG 21465	42.4	86.68
37.	*P. rhodesiae*	GCF_900105575	LMG 17764	50.2	86.62
38.	*P. salmasensis*	GCF_014268375	SWRI126	47.1	87.00
39.	*P. salomonii*	GCA_001730645	LMG 22120	49.4	87.29
40.	*P. simiae*	GCA_001730615	CCUG 50988	56.2	87.55
41.	*P. sivasensis*	GCA_013778505	P7	52.1	87.36
42.	*P. spelaei*	GCA_009724245	CCM 7893	40.4	84.92
43.	*P. synxantha*	GCF_001439725	DSM 18928	49.2	87.32
44.	*P. tolaasii*	GCA_002813445	NCPPB 2192	45.4	86.95
45.	*P. tritici*	GCF_014268275	SWRI145	52.3	87.46
46.	*P. trivialis*	GCA_001730655	LMG 21464	42.8	86.80
47.	*P. veronii*	GCF_001439695	DSM 11331	57.2	88.17

48.	*P. yamanorum*	GCA_900105735	LMG 27247	45.2	85.78

Table S2. Similarity of predicted gene products from the *nah*-, *sal*- and *alk*- gene clusters of *Pseudomonas* sp. OVF7 to selected homologs

OVF7	Strand[a]	GC%	Proposed function	No. of amino acids, OVF7/ relative[b]	% Amino Acid identity	Source microorganism	GeneBank accession number[c]
				n-alkane catabolic genes			
alkT	-	47.8	Rubredoxin-NAD(+) reductase	385/385	100	*Pseudomonas veronii* 7-41 (pCPC7-41 plasmid)	UHH01061.1
				385/385	100	*Pseudomonas veronii* VI4T1	OPK05674.1
				385/385	100	*Pseudomonas putida* P1	CAB69078.1
				385/385	92	*Pseudomonas putida* strain GPo1 (OCT plasmid)	CAB54063.1
				385/385	93	*Stutzerimonas stutzeri* strain DN36	UVO16372.1
alkS	-	45.4	*alk* gene regulator	883/883	100	*Pseudomonas veronii* 7-41 (pCPC7-41 plasmid)	UHH01060.1
				883/883	100	*Pseudomonas veronii* VI4T1	OPK05675.1
				883/883	100	*Pseudomonas putida* P1	CAB69079.1
				883/882	84	*Pseudomonas putida* strain GPo1 (OCT plasmid)	CAB54064.1
				883/882	97	*Stutzerimonas stutzeri* strain DN36	UVO16371.1
alkB	+	48.2	Alkane 1-monooxygenase	402/402	100	*Pseudomonas veronii* 7-41 (pCPC7-41 plasmid)	UHH01058.1
				402/402	100	*Pseudomonas veronii* VI4T1	OPK05677.1
				402/402	100	*Pseudomonas putida* P1	CAB51047.1
				402/401	92	*Pseudomonas putida* strain GPo1 (OCT plasmid)	CAB54050.1
				402/402	99	*Stutzerimonas stutzeri* strain DN36	UVO16370.1
alkF	+	43.5	Rubredoxin-1	134/134	100	*Pseudomonas veronii* 7-41 (pCPC7-41 plasmid)	UHH01057.1

				134/134	100	*Pseudomonas veronii* VI4T1	OPK05678.1
				134/134	100	*Pseudomonas putida* P1	CAB51048.1
				134/132	47	*Pseudomonas putida* strain GPo1 (OCT plasmid)	CAB54051.1
				134/131	98	*Stutzerimonas stutzeri* strain DN36	UVO16369.1
alkG	+	45.9	Rubredoxin-2	175/175	100	*Pseudomonas veronii* 7-41 (pCPC7-41 plasmid)	UHH01056.1
				175/175	100	*Pseudomonas veronii* VI4T1	OPK05679.1
				175/175	100	*Pseudomonas putida* P1	CAB51049.1
				175/173	64	*Pseudomonas putida* strain GPo1 (OCT plasmid)	CAB54052.1
				175/175	98	*Stutzerimonas stutzeri* strain DN36	UVO16368.1
alkH	+	44.1	Aldehyde dehydrogenase	483/483	100	*Pseudomonas veronii* 7-41 (pCPC7-41 plasmid)	UHH01055.1
				483/483	100	*Pseudomonas veronii* VI4T1	OPK05680.1
				483/483	100	*Pseudomonas putida* P1	CAB51050.1
				483/483	81	*Pseudomonas putida* strain GPo1 (OCT plasmid)	CAB54053.1
				483/483	100	*Stutzerimonas stutzeri* strain DN36	UVO16367.1
alkJ	+	43.7	Alcohol dehydrogenase	552/552	100	*Pseudomonas veronii* 7-41 (pCPC7-41 plasmid)	UHH01054.1
				552/552	100	*Pseudomonas veronii* VI4T1	OPK05681.1
				552/552	100	*Pseudomonas putida* P1	CAB51051.1
				552/558	86	*Pseudomonas putida* strain GPo1 (OCT plasmid)	CAB54054.1
				552/552	99	*Stutzerimonas stutzeri* strain DN36	UVO16366.1
alkK	+	45.5	long-chain-fatty-acid-CoA ligase	546/546	100	*Pseudomonas veronii* 7-41	UHH01053.1

						(pCPC7-41 plasmid)	
				546/546	100	*Pseudomonas veronii* VI4T1	OPK05682.1
				546/546	99	*Pseudomonas putida* P1	CAB69080.1
				546/546	81	*Pseudomonas putida* strain GPo1 (OCT plasmid)	CAB54055.1
				546/546	98	*Stutzerimonas stutzeri* strain DN36	UVO16365.1
alkL	+	41	Outer membrane beta-barrel protein	230/230	100	*Pseudomonas veronii* 7-41 (pCPC7-41 plasmid)	UHH01052.1
				230/230	100	*Pseudomonas veronii* VI4T1	OPK05683.1
				230/230	100	*Pseudomonas putida* P1	CAB69081.1
				230/230	80	*Pseudomonas putida* strain GPo1 (OCT plasmid)	CAB54056.1
				230/230	98	*Stutzerimonas stutzeri* strain DN36	UVO16364.1
naphthalene catabolic genes							
nahX	-	62.6	heme-binding protein	143/146	100	*Pseudomonas veronii* 7-41 (pCPC7-41 plasmid)	UHH01047.1
				143/146	100	*Pseudomonas veronii* VI4T1	OPK05596.1
				143/140	72	*Pseudomonas putida* plasmid NAH7	WP_011475396 .1
				143/146	84	*Pseudomonas veronii* strain Pvy plasmid unnamed	WP_003292104 .1
				143/146	84	*Stutzerimonas stutzeri* strain J15	UUC84787.1
nahJ	-	57.8	4-oxalocrotonate tautomerase	63/63	100	*Pseudomonas veronii* 7-41 (pCPC7-41 plasmid)	UHH01046.1
				63/63	100	*Pseudomonas veronii* VI4T1	OPK05595.1
				63/63	85	*Pseudomonas putida* plasmid NAH7	WP_011475395 .1
				63/63	79	*Pseudomonas veronii* strain Pvy plasmid unnamed	WP_003292102 .1

				63/63	79	*Stutzerimonas stutzeri* strain J15	UUC84788.1
nahK	-	62.5	4-oxalocrotonate decarboxylase	264/264	100	*Pseudomonas veronii* 7-41 (pCPC7-41 plasmid)	UHH01045.1
				264/264	100	*Pseudomonas veronii* VI4T1	OPK05594.1
				264/264	98	*Pseudomonas putida* plasmid NAH7	WP_011475394 .1
				264/264	91	*Pseudomonas veronii* strain Pvy plasmid unnamed	WP_003292101 .1
				264/264	92	*Stutzerimonas stutzeri* strain J15	UUC84789.1
nahM	-	63.1	4-hydroxy-2-oxovalerate aldolase	346/346	100	*Pseudomonas veronii* 7-41 (pCPC7-41 plasmid)	UHH01044.1
				346/346	100	*Pseudomonas veronii* VI4T1	OPK05593.1
				346/346	96	*Pseudomonas putida* plasmid NAH7	WP_011475393 .1
				346/346	94	*Pseudomonas veronii* strain Pvy plasmid unnamed	WP_003450283 .1
				346/346	94	*Stutzerimonas stutzeri* strain J15	UUC84790.1
nahO	-	64.7	Acetaldehyde dehydrogenase	307/307	100	*Pseudomonas veronii* 7-41 (pCPC7-41 plasmid)	UHH01043.1
				307/307	100	*Pseudomonas veronii* VI4T1	OPK05592.1
				307/307	97	*Pseudomonas putida* plasmid NAH7	WP_011475392 .1
				307/307	94	*Pseudomonas veronii* strain Pvy plasmid unnamed	WP_009397182 .1
				307/307	94	*Stutzerimonas stutzeri* strain J15	UUC84791.1
nahL	-	64.5	2-oxypent-4-enoate hydratase	261/261	99	*Pseudomonas veronii* 7-41 (pCPC7-41 plasmid)	UHH01042.1
				261/261	100	*Pseudomonas veronii* VI4T1	OPK05591.1
				261/261	97		

						Pseudomonas putida plasmid NAH7	WP_011475391.1
				261/261	87	*Pseudomonas veronii* strain Pvy plasmid unnamed	WP_009397181.1
				261/261	89	*Stutzerimonas stutzeri* strain J15	UUC84792.1
nahN	-	65.3	2-hydroxymuconic semialdehyde hydrolase	297/289	100	*Pseudomonas veronii* 7-41 (pCPC7-41 plasmid)	UHH01041.1
				297/293	99	*Pseudomonas veronii* VI4T1	OPK05599.1
				297/293	79	*Pseudomonas putida* plasmid NAH7	WP_011475390.1
				297/287	83	*Pseudomonas veronii* strain Pvy plasmid unnamed	WP_009397180.1
				297/282	82	*Stutzerimonas stutzeri* strain J15	UUC84793.1
nahI	-	63.7	2-Hydroxymuconic semialdehyde dehydrogenase	486/486	100	*Pseudomonas veronii* 7-41 (pCPC7-41 plasmid)	UHH01040.1
				486/486	100	*Pseudomonas veronii* VI4T1	OPK05590.1
				486/486	94	*Pseudomonas putida* plasmid NAH7	WP_011475389.1
				486/486	91	*Pseudomonas veronii* strain Pvy plasmid unnamed	WP_009397179.1
				486/486	92	*Stutzerimonas stutzeri* strain J15	UUC84794.1
nahH	-	52.5	Catechol 2,3-dioxygenase	307/307	100	*Pseudomonas veronii* 7-41 (pCPC7-41 plasmid)	UHH01039.1
				307/307	100	*Pseudomonas veronii* VI4T1	OPK05589.1
				307/307	87	*Pseudomonas putida* plasmid NAH7	WP_011475388.1
				307/307	89	*Pseudomonas veronii* strain Pvy plasmid unnamed	WP_009397178.1
				307/307	89	*Stutzerimonas stutzeri* strain J15	UUC84795.1

nahT	-	53.6	Chloroplast ferredoxin-like protein	106/106	100	*Pseudomonas veronii* 7-41 (pCPC7-41 plasmid)	UHH01038.1
				106/106	100	*Pseudomonas veronii* VI4T1	OPK05588.1
				106/108	78	*Pseudomonas putida* plasmid NAH7	WP_011475387.1
				106/112	88	*Pseudomonas veronii* strain Pvy plasmid unnamed	WP_003292091.1
				106/112	87	*Stutzerimonas stutzeri* strain J15	UUC84796.1
nahG	-	58.6	Salicylate 1-monooxygenase	434/434	100	*Pseudomonas veronii* 7-41 (pCPC7-41 plasmid)	UHH01037.1
				434/434	100	*Pseudomonas veronii* VI4T1	OPK05587.1
				434/434	86	*Pseudomonas putida* plasmid NAH7	WP_011475386.1
				434/437	86	*Pseudomonas veronii* strain Pvy plasmid unnamed	WP_043221467.1
				434/437	86	*Stutzerimonas stutzeri* strain J15	UUC84797.1
nahR	+	56.6	LysR-type transcriptional regulator	300/300	100	*Pseudomonas veronii* 7-41 (pCPC7-41 plasmid)	UHH01036.1
				300/300	100	*Pseudomonas veronii* VI4T1	OPK05586.1
				300/374	82	*Pseudomonas putida* plasmid NAH7	WP_011475385.1
				300/300	95	*Pseudomonas veronii* strain Pvy plasmid unnamed	WP_003292087.1
				300/300	95	*Stutzerimonas stutzeri* strain J15	UUC84798.1
nahAa	+	54.2	Naphthalene 1,2-dioxygenase reductase	328/328	100	*Pseudomonas veronii* 7-41 (pCPC7-41 plasmid)	UHH01033.1
				328/328	100	*Pseudomonas veronii* VI4T1	OPK03992.1
				328/328	84	*Pseudomonas putida* plasmid NAH7	WP_011475375.1

				328/328	89	*Pseudomonas veronii* strain Pvy plasmid unnamed	WP_155678975.1
				328/328	90	*Stutzerimonas stutzeri* strain J15	UUC84813.1
nahAb	+	47.7	Naphthalene 1,2-dioxygenase ferredoxin	108/108	100	*Pseudomonas veronii* 7-41 (pCPC7-41 plasmid)	UHH01032.1
				108/108	100	*Pseudomonas veronii* VI4T1	OPK03993.1
				108/107	89	*Pseudomonas putida* plasmid NAH7	WP_011475376.1
				108/104	89	*Pseudomonas veronii* strain Pvy plasmid unnamed	WP_009399901.1
				108/118	88	*Stutzerimonas stutzeri* strain J15	UUC84812.1
nahAc	+	49.5	Naphthalene 1,2-dioxygenase large subunit	449/449	100	*Pseudomonas veronii* 7-41 (pCPC7-41 plasmid)	UHH01031.1
				449/449	100	*Pseudomonas veronii* VI4T1	OPK03994.1
				449/449	94	*Pseudomonas putida* plasmid NAH7	WP_011475377.1
				449/449	93	*Pseudomonas veronii* strain Pvy plasmid unnamed	WP_155678976.1
				449/449	93	*Stutzerimonas stutzeri* strain J15	UUC84811.1
nahAd	+	50.2	Naphthalene 1,2-dioxygenase small subunit	193/193	100	*Pseudomonas veronii* 7-41 (pCPC7-41 plasmid)	UHH01030.1
				193/193	100	*Pseudomonas veronii*VI4T1	OPK03995.1
				193/193	85	*Pseudomonas stutzeri*	WP_020307249.1
				193/193	83	*Pseudomonas putida* plasmid NAH7	WP_011475378.1
				193/193	83	*Pseudomonas veronii* strain Pvy plasmid unnamed	WP_141123763.1
				193/193	85	*Stutzerimonas stutzeri* strain J15	UUC84810.1
nahB	+	49.4	Naphthalene *cis*-dihydrodiol	259/259	100	*Pseudomonas veronii* 7-41	UHH01029.1

			dehydrogenase			(pCPC7-41 plasmid)	
				259/259	100	*Pseudomonas veronii* VI4T1	OPK03996.1
				259/259	93	*Pseudomonas veronii* strain Pvy plasmid unnamed	WP_141123764.1
				259/259	91	*Pseudomonas putida* plasmid NAH7	WP_011475379.1
				259/275	91	*Pseudomonas putida* AK5	AFM52767.1
				259/259	93	*Stutzerimonas stutzeri* strain J15	UUC84809.1
nahF	+	53.6	Salicylaldehyde dehydrogenase	483/483	100	*Pseudomonas veronii* 7-41 (pCPC7-41 plasmid)	UHH01028.1
				483/483	100	*Pseudomonas veronii* VI4T1	OPK03997.1
				483/483	91	*Pseudomonas putida* plasmid NAH7	WP_011475380.1
				483/483	92	*Pseudomonas veronii* strain Pvy plasmid unnamed	WP_155678977.1
				483/483	93	*Pseudomonas* sp. MPDS	WP_173407259.1
				483/483	93	*Stutzerimonas stutzeri* strain J15	UUC84808.1
nahC	+	50.5	1,2-dihydroxynaphthalene dioxygenase	302/302	100	*Pseudomonas veronii* 7-41 (pCPC7-41 plasmid)	UHH01027.1
				302/302	92	*Pseudomonas putida* plasmid pDTG1	WP_011117404.1
				302/302	92	*Pseudomonas veronii* strain Pvy plasmid unnamed	WP_141123765.1
				302/302	100	*Pseudomonas veronii*VI4T1	OPK03998.1
				302/302	89	*Stutzerimonas stutzeri* strain J15	UUC84807.1
nahQ	+	48.1	Outer membrane beta-barrel protein	211/211	99	*Pseudomonas veronii* 7-41 (pCPC7-41 plasmid)	UHH01026.1
				211/180	82	*Pseudomonas frederiksbergensis*	WP_235864979.1

						strain AS1 plasmid unnamed	
				211/211	80	*Pseudomonas veronii* strain Pvy plasmid unnamed	WP_012727770.1
				211/215	79	*Pseudomonas putida* plasmid pDTG1	WP_228409390.1
				211/210	77	*Pseudomonas putida* plasmid NAH7	WP_011475382.1
				211/211	99	*Pseudomonas veronii* VI4T1	OPK03999.1
nahE	+	52	1, 2-dihydroxybenzylpyruvate aldolase	334/334	100	*Pseudomonas veronii* 7-41 (pCPC7-41 plasmid)	UHH01025.1
				334/334	92	*Pseudomonas stutzeri* AN10	AAD02141.1
				334/331	89	*Pseudomonas putida* plasmid NAH7	WP_011475383.1
				334/334	100	*Pseudomonas veronii* VI4T1	OPK04000.1
				334/334	92	*Stutzerimonas stutzeri* strain J15	UUC84806.1
nahD	+	46.6	2-hydroxychromene-2-carboxylate isomerase	200/200	100	*Pseudomonas veronii* 7-41 (pCPC7-41 plasmid)	UHH01024.1
				200/203	78	*Pseudomonas putida* plasmid NAH7	WP_011475384.1
				200/199	78	*Pseudomonas veronii* strain Pvy plasmid unnamed	WP_011117407.1
				200/200	100	*Pseudomonas veronii* VI4T1	OPK04001.1
				200/222	77	*Stutzerimonas stutzeri* strain J15	UUC84805.1

[a] Coding chain designation: -, complementary strand; +, direct strand.
[b] Number of amino acids in OVF7 CDS/number in the closest relative.
[c] GenBank accession number of the closest relative protein.

microorganisms
MDPI

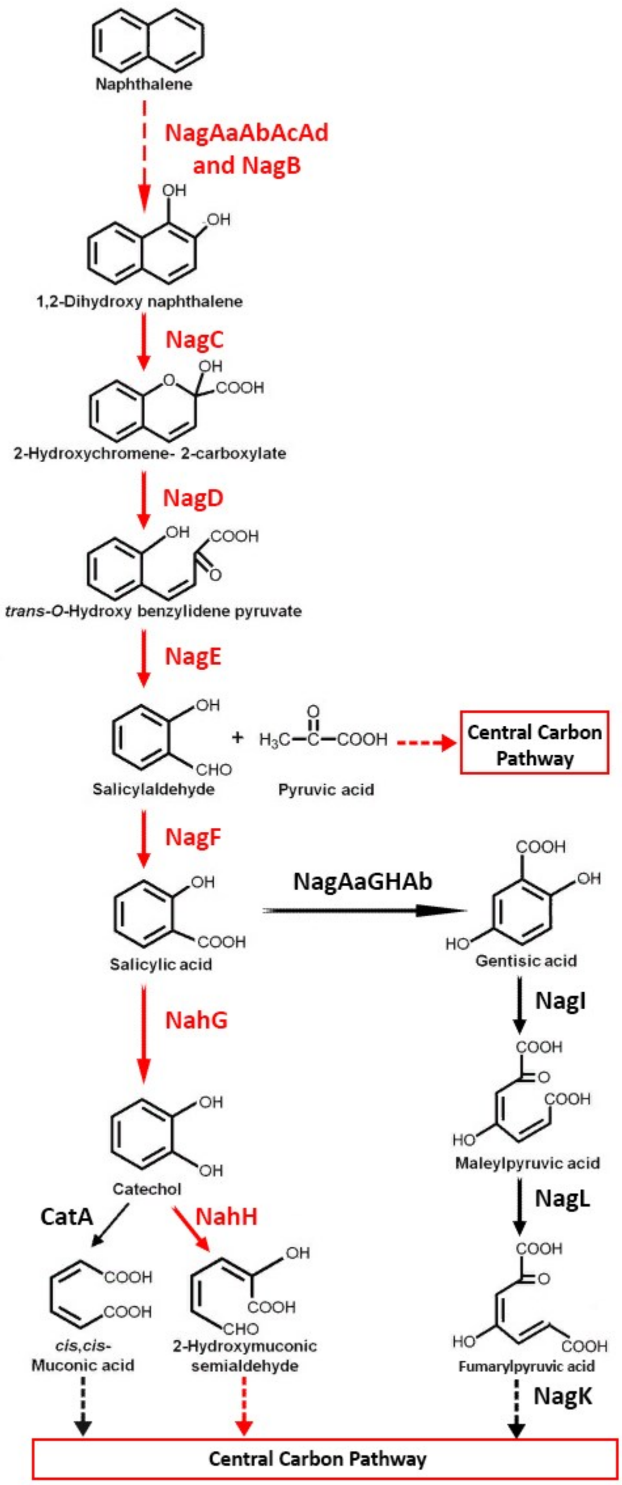

Figure S1. Scheme of naphthalene catabolic pathway via catechol (red arrows) and gentisic acid (black arrows). The enzymes involved in naphthalene catabolism are naphthalene 1.2-dioxygenase (NagAaAbAcAd), *cis*-naphthalene dihydrodiol dehydrogenase (NagB), 1,2-dihydroxynaphthalene dioxygenase (NagC), 2-hydroxy-2H-chromene-2-carboxylate isomerase (NagG), *trans*-ohydroxybenzylidenepyruvate hydratase-aldolase (NagE), salicylaldehyde dehydrogenase (NagF), salicylate hydroxylase (NahG), catechol 2,3-dioxygenase (NahH), catechol 1,2-dioxygenase (catA), salicylate 5-hydroxylase (NagGHAaAb), gentisate 1,2-dioxygenase (NagI), maleylpyruvate isomerase (NagL), and fumarylpyruvate hydrolase (NagK). The red color indicates the naphthalene degradation pathway by the *Pseudomonas sp.* strain OVF7 and the enzymes involved in its catabolism.

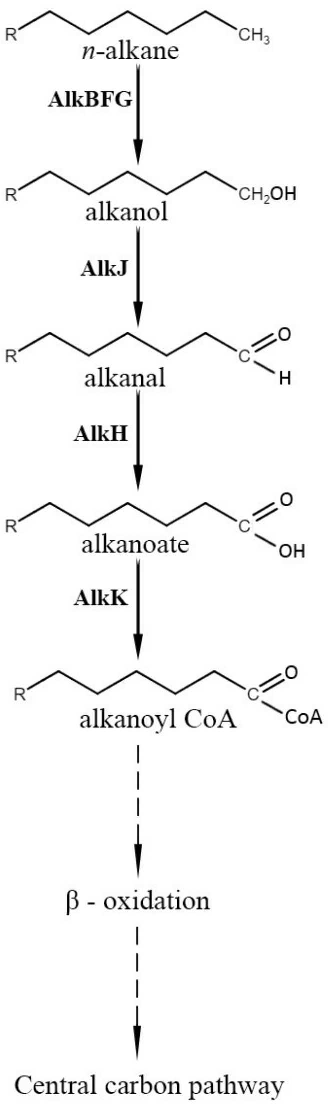

Figure S2. Scheme of *n*-alkane catabolic pathway. The enzymes involved in *n*-alkane catabolism are alkane monooxygenase (AlkBFG), alcohol dehydrogenase (AlkJ), aldehyde dehydrogenase (AlkH), and acetyl-CoA synthetase (AlkK).
